# Intrinsic Cellular Responses of Human Wharton’s Jelly Mesenchymal Stem Cells Influenced by O_2_-Plasma-Modified and Unmodified Surface of Alkaline-Hydrolyzed 2D and 3D PCL Scaffolds

**DOI:** 10.3390/jfb10040052

**Published:** 2019-11-18

**Authors:** Kewalin Inthanon, Wanida Janvikul, Siriwan Ongchai, Siriwadee Chomdej

**Affiliations:** 1 Department of Biotechnology, Faculty of Science and Technology, Thammasat University, Lampang 52190, Thailand; 2 National Metal and Materials Technology Center, Pathumthani 12120, Thailand; wanidaj@mtec.or.th; 3 Thailand Excellence Centre for Tissue Engineering and Stem Cells, Department of Biochemistry and the Center of Excellence for Innovation in Chemistry, Faculty of Medicine, Chiang Mai University, Chiang Mai 50200, Thailand; ongchai.submission@gmail.com; 4 Center of Excellence in Bioresources for Agriculture, Industry and Medicine Chiang Mai University, Chiang Mai 50200, Thailand

**Keywords:** polycaprolactone, oxygen plasma, Wharton’s Jelly Mesenchymal Stem Cells

## Abstract

Polycaprolactone (PCL), a hydrophobic-degradable polyester, has been widely investigated and extensively developed, to increase the biocompatibility for tissue engineering. This research was the first trial to evaluate the intrinsic biological responses of human Wharton’s Jelly Mesenchymal Stem Cells (hWJMSCs) cultured on alkaline hydrolysis and low-pressure oxygen plasma modified 2D and 3D PCL scaffolds, without adding any differentiation inducers; this has not been reported before. Four types of the substrate were newly established: 2D plasma-treated PCL (2D-TP), 2D non-plasma-treated PCL (2D-NP), 3D plasma-treated PCL (3D-TP), and 3D non-plasma-treated PCL (3D-NP). Physicochemical characterization revealed that only plasma-treated PCL scaffolds significantly increased the hydrophilicity and % oxygen/carbon ratio on the surfaces. The RMS roughness of 3D was higher than 2D conformation, whilst the plasma-treated surfaces were rougher than the non-plasma treated ones. The cytocompatibility test demonstrated that the 2D PCLs enhanced the initial cell attachment in comparison to the 3Ds, indicated by a higher expression of focal adhesion kinase. Meanwhile, the 3Ds promoted cell proliferation and migration as evidence of higher cyclin-A expression and filopodial protrusion, respectively. The 3Ds potentially protected the cell from apoptosis/necrosis but also altered the pluripotency/differentiation-related gene expression. In summary, the different configuration and surface properties of PCL scaffolds displayed the significant potential and effectiveness for facilitating stem cell growth and differentiation in vitro. The cell–substrate interactions on modified surface PCL may provide some information which could be further applied in substrate architecture for stem cell accommodation in cell delivery system for tissue repair.

## 1. Introduction

This research was the first to reveal the effects of alkaline hydrolysis and oxygen-plasma modification surface of 2D and 3D polycaprolactone (PCL) scaffolds on cytocompatibility and cell-substrate mechanotransduction through human Wharton’s Jelly Mesenchymal Stem Cells (hWJMSCs). Although the PCL has been extensively applied in tissue engineering for many years, worldwide laboratories continuously developed the well-designed biomaterials, to support and facilitate cell growth and propagation. Unfortunately, not all biomaterials can support all cell types, including stem cells. The establishment of a cell-friendly material facilitating cell adhesion, propagation, homing, and accommodating without using any growth factor or inducing molecule might offer some advantages for a new generation of multidisciplinary cell-based tissue engineering.

Improvement of a biocompatible substrate for regenerative medicine has been the focus of intensive research efforts for several decades [1]. Material composition and fabrication technique must be carefully selected according to the target cell type and ultimate application. The surface architecture of these materials has been refined to micro- and nanoscales in order to mimic specific cell niches. In cell-based therapy, the biomaterial must permit cell attachment, proliferation, homeostasis, and tissue formation [2,3,4]. However, many limitations of previous biomaterial support platforms have been encountered, including an inability to control cell growth and differentiation [5]. In addition, most regenerative medicine and tissue engineering techniques are based on exogenous cell-culture systems that may induce graft versus host disease. In response, endogenous regenerative medicine has been developed so that the patient’s own cells or tissues are used [6] to support and promote cell proliferation, survival, and differentiation, thereby increasing intrinsic regenerative capacity after transplantation [7]. 

Polycaprolactone (PCL) is a well-known biocompatibility and biodegradable polymer. To improve the overall properties of PCL, various methods have been invented [8], such as plasma or laser surface modification, as well as acid-alkaline leaching, which altered the native topography of the PCL surface conducting to significantly enhance cell attachment and proliferation [4]. Substrate geometry, topography, and surface properties potentially lead to cellular mechanotransduction involving in the determination of stem cell behavior on PLC and other materials [9]. For instance, 2D scaffolds are generally considered more suitable for tissue layer construction, such as for skin and the cornea, while 3D scaffolds may be more applicable for complex tissue formation by permitting the coordinated cell–cell and cell–matrix interactions required for appropriate cell migration and differentiation [9]. Recently, PCL was invented to advance utilities in tissue engineering, such as a novel injectable self-cross-linkable hyper-branched poly(ε-caprolactone) (hyPCL), which was fabricated for extensive medicinal applications, including bone defect repair [10]. Another study revealed that the surface-modified porous 3D scaffolds from PCL and graphene by a coating of polydopamine and arginylglycylaspartic acid have been used for severe nerve injury reconstruction and have led to many advantages in neuro-regenerative medicine [11]. 

To improve the biocompatibility of the scaffolds, surface modification has been widely applied, including alkaline and plasma treatment. PCL films and other grafting PCL scaffolds were found to increase their surface hydrophobicity after partial hydrolysis by sodium hydroxide [12,13,14]. The alkaline treatment has been invented to various types of biodegradable materials since the surface morphology alteration cannot be observed after treatment [15,16]. Oxygen plasma treatment also increases the hydrophilicity of the scaffold surface and plays an important role in the alteration of surface roughness. The oxygen plasma added some polar functional groups on the PCL surface [17], consequently resulting in better cell attachment and proliferation [1,4,18]. Such surface treatments provided a better substrate for many kinds of cells, such as chondrocytes, smooth muscle cells, and mesenchymal stem cells for tissue engineering application.

The aim of the current study was to develop and evaluate a tunable biomaterial with the potential for stem cell modulation and endogenous regenerative medicine applications. The alkaline-hydrolyzed PCL was fabricated by a high-pressure supercritical CO_2_ technique, followed by low-pressure oxygen plasma surface modification, as described by Kosorn et al. (2012) [19]. PCL scaffolds were constructed as 2D and 3D platforms and assessed for biocompatibility by evaluating attachment, proliferation, apoptosis, stem maintenance, and differentiation of the human Wharton’s jelly mesenchymal stem cell (hWJMSC) line.

## 2. Materials and Methods 

### 2.1. Preparation of Plasma Modified 2D and 3D PCL Scaffolds

**Figure 1:**
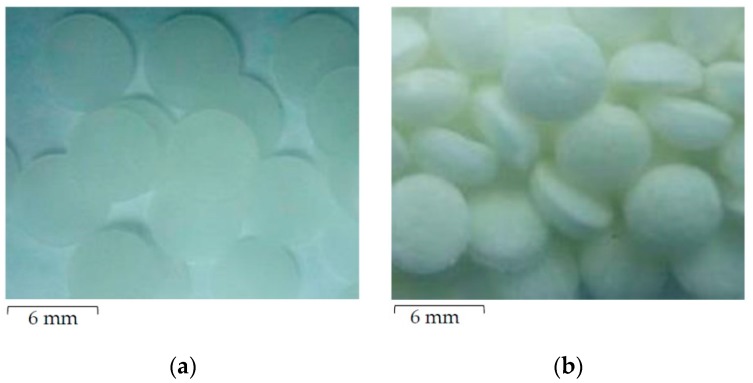
The appearance of finely cut circular polycaprolactone (PCL) scaffolds of (**a**) the 2-dimensional (2D) flat sheet and (**b**) the 3-dimensional (3D) sponge disc.

**Figure 2:**
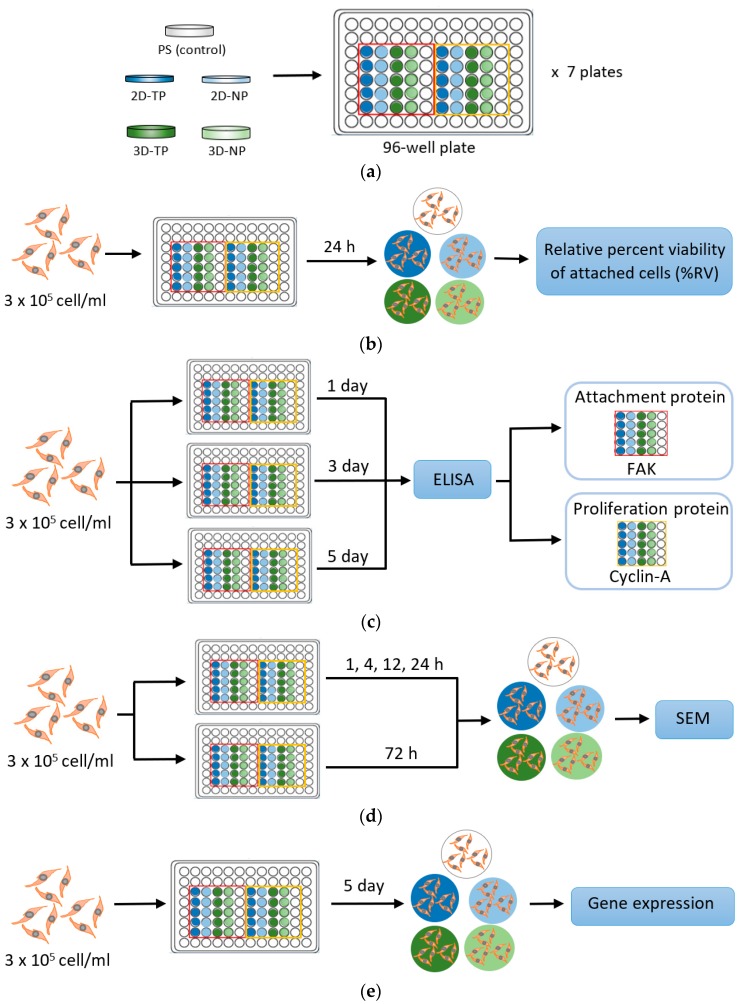
Simple diagrams exhibiting a protocol of each experiment performed in this research. (**a**) Polycaprolactone (PCL) scaffold preparation; (**b**) Initial cell attachment assay; (**c**) Cell proliferation and attachment assays by enzyme-linked immunosorbent assay (ELISA); (**d**) Observation of cellular attachment under scanning electron microscopy (SEM); (**e**) Gene expression by semi-quantitative polymerase chain reaction (PCR).

Scaffolds of PCL in 2- and 3-dimensional (2D and 3D) forms were obtained from the National Metal and Materials Technology (MTEC, Bangkok, Thailand). Fabrication and oxygen plasma surface modifications were performed as previously described by Kosorn et al. (2012) [19]. Briefly, the 2D PCL scaffolds were prepared by hydrolysis of PCL pellets (Sigma-Aldrich, St. Louis, MO, USA) in 6 N of NaOH, at 50 °C, for 5 h, followed by soaking in deionized water and freeze-drying overnight. For the 3D scaffold preparation, the PCL pellets were loaded into a cylindrical vessel, heated at 60 °C for 10 min, filled with CO_2_ at 15 MPa, and then soaked in deionized water for 3 h. Both 2D and 3D PCL scaffolds were then treated with pure oxygen plasma, using a low-pressure radiofrequency discharge plasma cleaner (model PDC-002, Harrick, Ithaca, NY, USA) at 30 W for 30 min. In this study, plasma-treated 2D and 3D PCL scaffolds are abbreviated as 2D-TP and 3D-TP, while untreated 2D and 3D PCL scaffolds are termed 2D-NP and 3D-NP, respectively. All types of scaffolds were finely cut into small circular pieces that were 6 mm in diameter for each well of 96-well plate (Figure 1). The 2D scaffold was similar to a flat sheet with a thickness of 0.5 mm (Figure 1a), whilst the 3D looked like a sponge disc with a thickness of 2.0 mm (Figure 1b). To sterilize the scaffolds for cell culture, each side was exposed for 15 min to standard UV light in a biosafety cabinet. The sterile circular piece was then immersed into the bottom of a well in a 96-well plate, washed by 1X phosphate buffer saline (PBS) twice, and then filled up with the complete medium mixture, for at least 30 min. This immersion steps aimed to avoid scaffold floating from the bottom of the culture plate since the air bubbles in the scaffold were fully replaced with the complete medium. After that, the scaffold was ‘ready to use’ for further cell culture experiment. The protocol diagram is provided in Figure 2a. 

### 2.2. PCL Scaffold Characterization

The physicochemical properties of the scaffold were first characterized. The surface morphology of the scaffolds was photomicrographed by scanning electron microscopy (SEM, Hitachi, Japan). Surface topology of the scaffold was visualized and analyzed by using Gwyddion Software Version 2.53 (Brno, Czech Republic), resulting in root-mean-square (RMS) roughness. The hydrophilicity of the scaffold was measured from the water contact angle, using an optical bench-type contact angle goniometer (Ramé-Hart, Succasunna, NJ, USA). The chemical composition of the surface was evaluated through X-ray photoelectron spectroscopy (XPS, Kratos Analytical, Manchester, UK), and the data was presented as the percent oxygen/carbon ratio (% O/C ratio).

### 2.3. Standard Cell Culture

The research was conducted in accordance with the Declaration of Helsinki, and the protocol was approved by the Ethics Committee of Faculty of Medicine, Chiang Mai University (Ethics approval number: EXEMPTION-6302/2019). The hWJMSC line was obtained from The Human and Animal Cell Culture Research Unit, Department of Biology, Faculty of Science, Chiang Mai University, Thailand. The cells were maintained and propagated in a complete medium containing Dulbecco’s modified Eagle medium (DMEM) supplemented with 10% (v/v) fetal bovine serum (FBS) and 2 ng/mL of basic fibroblast growth factor (bFGF) (all from Gibco, Waltham, MA, USA). Cells were incubated at 37 °C, under an atmosphere with 95% humidity and 5% CO_2_. The medium was replaced every 2–3 days. At 70% confluence, cells were subcultured by trypsinization.

### 2.4. Indirect Cell Viability Assay

The hWJMSCs at a density 3 × 10^5^ cell/mL were seeded onto the PCL scaffolds and maintained for 1, 3, or 5 days under standard conditions. The number and viability of attached cells from each sample were evaluated indirectly from the total cDNA. To perform the assay, the total RNA was extracted from the 1-, 3-, and 5-d cultured cells on the 2D and 3D PCL scaffolds, using an RNA extraction kit (NucleoSpin^®^ RNAII, Fisher Scientific, Dublin, Ireland). The cDNA was synthesized from mRNA via a reverse-transcriptase reaction, using a Phusion^®^ RT-PCR kit (Thermo Scientific, Waltham, MA, USA). The cDNA solution was read at an absorbance of 280 nm, using a NanoDrop^®^1000 (Thermo Scientific, Waltham, MA, USA). The absorbance values were then converted to total cDNA concentrations and were represented as percent relative cell viability (% RV), compared with the PS. Each sample was set up in five replicates. The protocol diagram is provided in Figure 2b.

### 2.5. Cell Proliferation and Attachment Assays by ELISA

The cells were suspended in medium and seeded at the density 3 × 10^5^ cell/mL onto the PCL scaffolds or polystyrene as control and maintained for 1, 3, or 5 days in culture, followed by analyses of cell proliferation and attachment by enzyme-linked immunosorbent assay (ELISA). Focal adhesion kinase (FAK) and cyclin-A protein expression levels were used as markers for cell proliferation and attachment, respectively. The assays were performed as described by Inthanon et al. (2016) [20]. Briefly, cells were fixed in 4% (v/v) formaldehyde for 30 min at room temperature and then washed three times (5 min/wash) with ice-cold washing buffer (0.05% (v/v) Tween 20 in 1X PBS), with gentle rocking. The samples were subsequently immersed in quenching buffer containing 1X PBS, 1% (v/v) H_2_O_2_, and 0.1% (v/v) sodium azide for 20 min and then in blocking solution containing 5% (v/v) bovine serum albumin (BSA) for 1 h. Diluted anti-FAK and anti-cyclin-A primary antibodies were added, drop-wise, onto the samples and incubated at 4 °C overnight. A horseradish peroxidase (HRP)-labeled secondary antibody was added onto each sample for 1 h before applying the HRP substrate, o-phenylenediamine dihydrochloride (OPD) (all purchased from Sigma-Aldrich, St. Louis, MO, USA). The absorbance was read at 492 nm on a microplate reader. The absorbance values were then calculated to percent relative expression (% RE) per cell in comparison to PS. Five independent replicates were performed for each sample. The protocol diagram is provided in Figure 2c.

### 2.6. Observation of Cellular Attachment under Scanning Electron Microscopy (SEM)

Attached cells were fixed in 2.5% (w/v) glutaraldehyde (Sigma-Aldrich, St. Louis, MO, USA) for 20 min after 1, 4, 12, 24, or 72 h in culture. The samples were washed three times with PBS (5 min per wash), with gentle rocking, dehydrated in graded ethanol solutions, and then air-dried at room temperature. The samples were set onto copper stubs and coated with gold particles in a sputter unit (SPITM Module Sputter/Carbon Coater system, SPI^®^ supplies, West Chester, PA, USA). Cell morphology on both 2D and 3D PCL scaffolds was observed under SEM. The protocol diagram was provided in Figure 2d.

### 2.7. Gene Expression by Semi-Quantitative Polymerase Chain Reaction (PCR)

A 3 × 10^5^ cell/mL suspension of cells was seeded on 2D PLC scaffolds, 3D PCL scaffolds, or PS and then maintained in culture for 5 days prior to RNA extraction. In this experiment, the PS control was divided into two subsets: 1-day and 5-day cultures, which are abbreviated as 1d-ctrl and 5d-ctrl, respectively. Total RNA extraction and first strand cDNA synthesis were performed by using an RNA extraction kit (NucleoSpin^®^ RNAII, Fisher Scientific, Dublin, Ireland). First strand cDNA was synthesized from mRNA via a reverse-transcriptase reaction, using a Phusion^®^ RT-PCR kit (Thermo Scientific, Waltham, MA, USA). The cDNA concentration was measured by using NanoDrop^®^1000 (Thermo Scientific, Waltham, MA, USA), prior, to adjust to a working concentration. The cDNA (100 ng per sample) was used as the template for gene expression assessment by semi-quantitative PCR. Two gene groups were examined: (i) The apoptosis/necrosis-related genes *caspase 8*, *Apaf-1*, *Bcl-2*, and *PARP-1*; and (ii) the pluripotency/differentiation-related genes *SSEA-4*, *NES* (encoding *nestin*), *COL2A1*, and *PPAR-2*. *GAPDH* was used as the internal control for all reactions. The primer sequences (with accession numbers) for each gene are shown in Table 1. PCR products were electrophoresed on 1% (w/v) agarose gels at 75 V for 45 min, and bands were photographed under a UV-transilluminator. Gene expression was indirectly quantified as band intensity, using the ImageJ software (NIH, version 1.52r, Bethesda, MD, USA). Expression levels were normalized to *GAPDH* expression and then 1d-ctrl. Each experimental condition was repeated five times. The protocol diagram is provided in Figure 2e.

### 2.8. Statistical Analysis

All data are presented as mean ± standard deviation (SD). Treatment group means were compared by student’s t-test and ANOVA for independent or paired samples, respectively. A *p*-value ≤ 0.05 was considered statistically significant for all tests. The difference symbol displaying in the histogram (*, **, I, II, α, β) indicated the statistical difference individually described in each figure.

## 3. Results

### 3.1. Physicochemical Properties of 2D and 3D PCL Scaffolds

**Figure 3:**
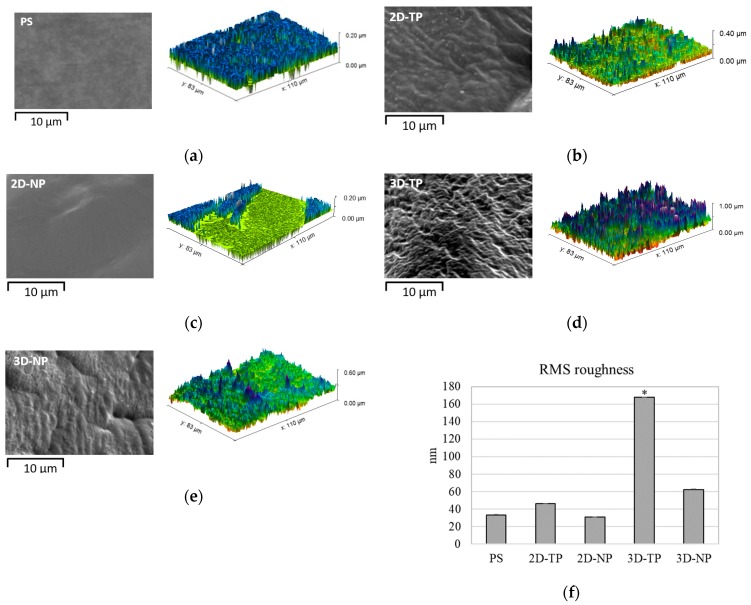
SEM (black-and-white) and AFM (color) visualization of surface topology and roughness for: (**a**) PS, (**b**) 2D-PCL, (**c**) 2D-NP, (**d**) 3D-TP, and (**e**) 3D-NP. (**f**) The RMS roughness data was analyzed by Gwyddion Software. An asterisk (*) next to the RMS values represented statistical differences (*p* ≤ 0.05) in roughness (nm) among the scaffold types.

The SEM picture displayed difference surface topography among the type of scaffolds, while the AFM visualized and analyzed the RMS roughness, and then the data was plotted (Figure 3). The roughest surface was observed in 3D-TP, with the significant highest amplitude (approximately 160 nm) (Figure 3d,f). The 3D-NP also significantly displayed the different roughness from PS control (approximately 60 nm) (Figure 3e,f). However, there was equivalent RMS roughness among PS, 2D-TP, and 2D-NP (Figure 3a–c,f). 

**Figure 4:**
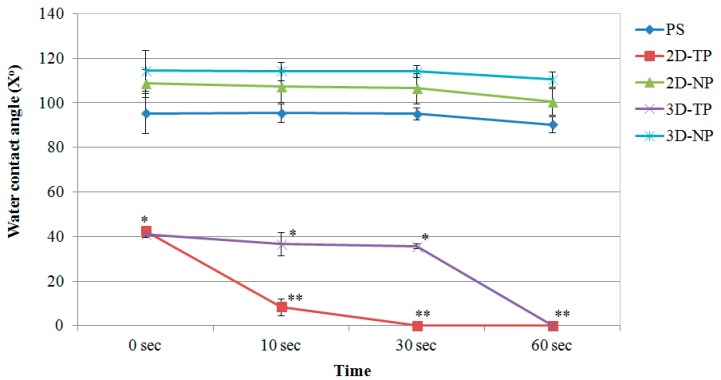
Water contact angle of membrane surfaces displayed as mean of degree (X°) ± SD at different time points. Asterisks (*, **) represented statistical differences (*p* ≤ 0.05) of X° among the scaffold samples at each time point.

The surface hydrophilicity of the PCL scaffolds increased in comparison to PS, 2D-NP, and 3D-NP after oxygen plasma surface treatment, observed from a decrease in the water contact angle (X°) at 0 s (Figure 4). 3D-NP exhibited the highest water contact angle, followed by 2D-NP and PS. No significant differences were found between the non-plasma-treated PCLs and PS. However, the water contact angle of all materials declined over time. The smallest contact angle was observed for 2D-TP, which was completely wet at 30 s, compared with 60 s for 3D-TP.

**Figure 5:**
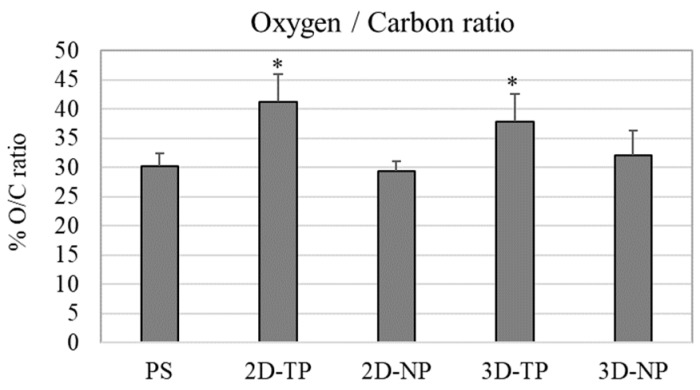
Percent of oxygen/carbon ratio (% O/C ratio) of the scaffold surface which was evaluated by XPS. An asterisk (*) on the top of the bar represented statistical differences (*p* ≤ 0.05) of % O/C ratio among the types of the scaffold.

From the chemical composition analysis in a number of oxygen and carbon atom by XPS, percent oxygen/carbon ratio (% O/C ratio) of the material surface was calculated and plotted (Figure 5). The highest % O/C ratio was observed in both 2D-TP and 3D-TP, which was statistically different from 2D-NP, 3D-NP, and PS.

### 3.2. Differences in Cell Viability, Attachment, and Proliferation on 2D and 3D Scaffolds

**Figure 6:**
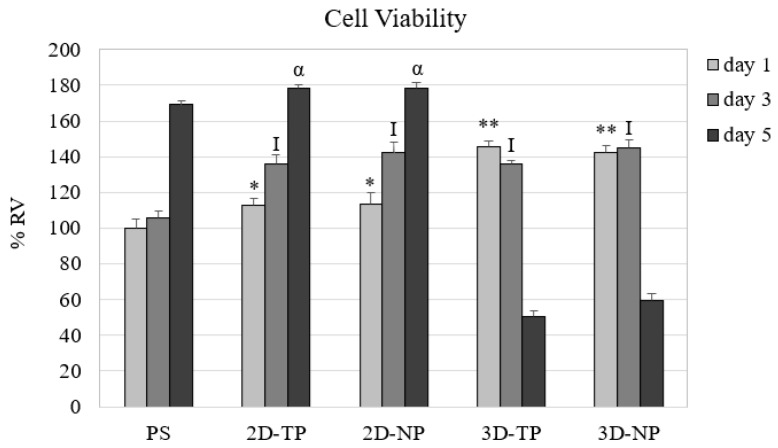
Percent relative cell viability (% RV) from the culture of day one, three, and five on different scaffolds. The symbols on the top of the bar represented significant differences (*p* ≤ 0.05) in % RV among the scaffolds on day one (*, **), three (I), and five (α).

After cell seeding for one, three, and five days, the number of viable attached cells was quantified indirectly from the total cDNA and converted to percent relative cell viability (% RV) (Figure 6). On day one, both types of 3D PCL exhibited the highest % RV, followed by the 2D PCLs. On day three, the cell viability on all PCL scaffolds converged. At day five, the 2D PCLs showed the highest % RV, while that of both 3D PCLs had dramatically reduced.

**Figure 7:**
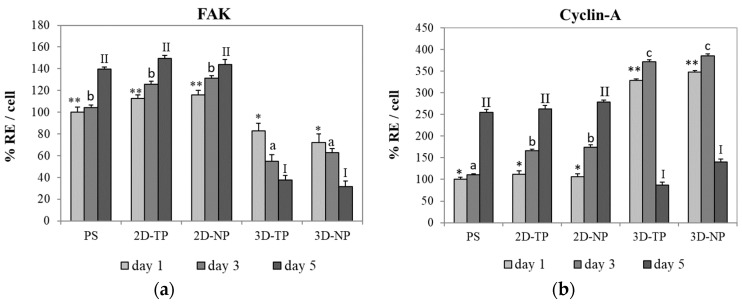
Percent relative expression per cell (% RE/cell) of Focal adhesion kinase: (**a**) FAK and (**b**) cyclin-A proteins by hWJMSC cultured on different scaffold types for days one, three, and five, as evaluated by enzyme-linked immunosorbent assay (ELISA). The symbols above the bars represented significant differences (*p* ≤ 0.05) in % RE among the scaffolds at day one (*, **), day three (a, b, c), and day five (I, II).

The capacity of PLC scaffolds that can support the attachment and proliferation of cells was evaluated by ELISA of FAK and cyclin-A protein expression, respectively. ELISA results showed that the hWJMSC cultures expressed higher FAK levels on 2D PCL scaffolds (both 2D-TP and 2D-NP) than on 3D scaffolds on all test days (Figure 7). The time course of the expression changes also differed among substrates. On 2D-TP and 2D-NP scaffolds, FAK expression increased progressively with time, while FAK expression decreased with time on 3D-TP and 3D-NP scaffolds. Further, the expression on 3D-TP scaffolds was markedly lower than on control PS and untreated 2D-TP scaffolds on all days. Conversely, cyclin-A expression on day one was substantially lower in cells growing on 2D-TP, 2D-NP, and control scaffolds than on 3D-TP and 3D-NP scaffolds. However, the expression on 2D and control scaffolds increased progressively, while expression on 3D scaffolds peaked on day three and decreased markedly thereafter (Figure 7). This expression pattern indicates that there was a high cell proliferation rate on the limited surface area of the 3D scaffolds until day three, each time cells reached confluence and cyclin-A is downregulated concomitant with proliferation rate (Figure 7).

### 3.3. Attached-Cell Morphology on Different Substrates

**Figure 8:**
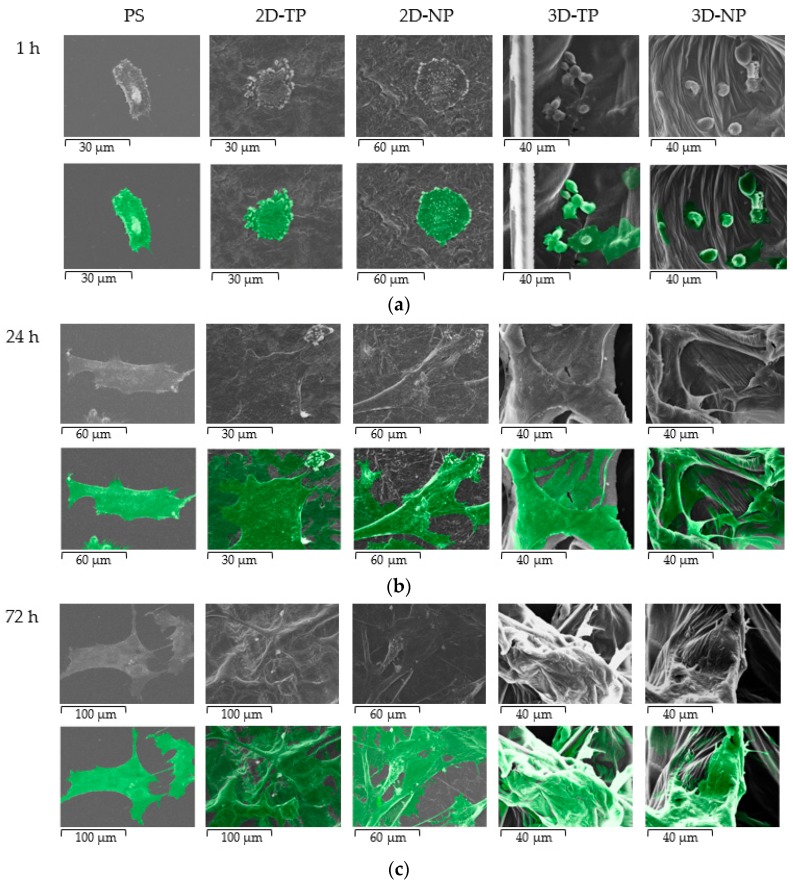
SEM Photomicrograph of hWJMSC morphology on PS, 2D-TP, 2D-NP, 3D-TP, and 3D-NP, at various times of culture: (**a**) 1 h, (**b**) 24 h, and (**c**) 72 h. The second row of each time point indicates the cell boundary and position on the scaffolds (exhibited in green).

**Figure 9:**
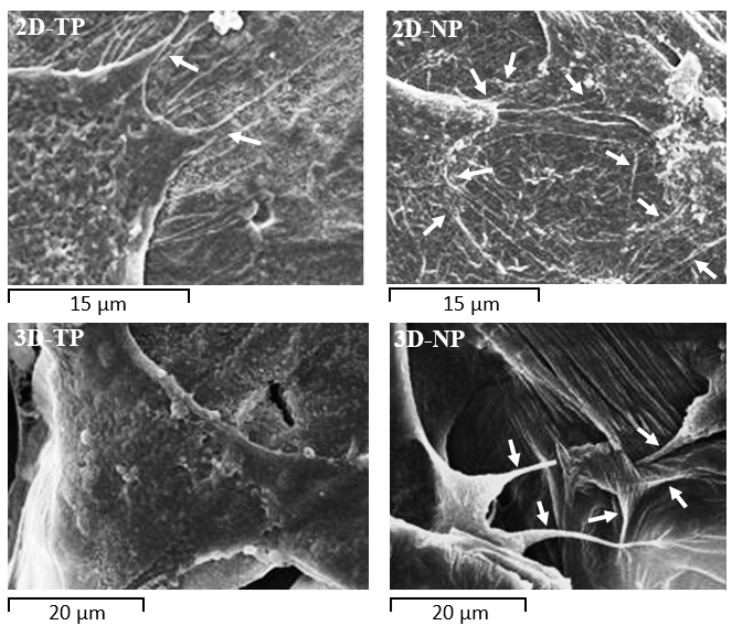
Filopodial protrusions (white arrows) extending from the cytoplasmic edge at 24 h of culture on PCL scaffolds. Cells on 2D-NP and 3D-NP scaffolds extended more numerous filopodia than cells on 2D-TP and 3D-TP, respectively.

The morphology of hWJMSC was monitored by SEM during culture for 72 h on the indicated substrate (Figure 8). These SEM pictures revealed a faster rate of cell attachment on 2D scaffolds and PS after cell seeding for an hour, as evidenced by a general flattener spindle shape and greater numbers of foot adherent pads (filopodia) compared to cells on other substrates (Figure 8a). Alternatively, cells on 3D scaffolds remained spherical at 1 h before attaching onto the surface and primarily exhibited irregular shapes and large adherent patches of cytoplasm around the nucleus at 24 h (Figure 7a,b on 3D-TP and 3D-NP column). At 72 h, there were more cells attached on the 2D PCL then 3D PCL and PS, respectively (Figure 8c). In addition, cells growing on oxygen-plasma treated PLC scaffolds either on 2D and 3D exhibited more numerous filopodial structures, observed from 24 h (Figure 9). However, more cells on 3D scaffolds exhibited irregular shapes and undefined cytoplasmic edges compared to cells on 2D PCL scaffolds.

### 3.4. Shifts in Apoptosis/Necrosis-Related and Pluripotency/Differentiation-Related Gene Expression on Different Substrates

#### 3.4.1. Apoptosis/Necrosis-Related Genes

**Figure 10:**
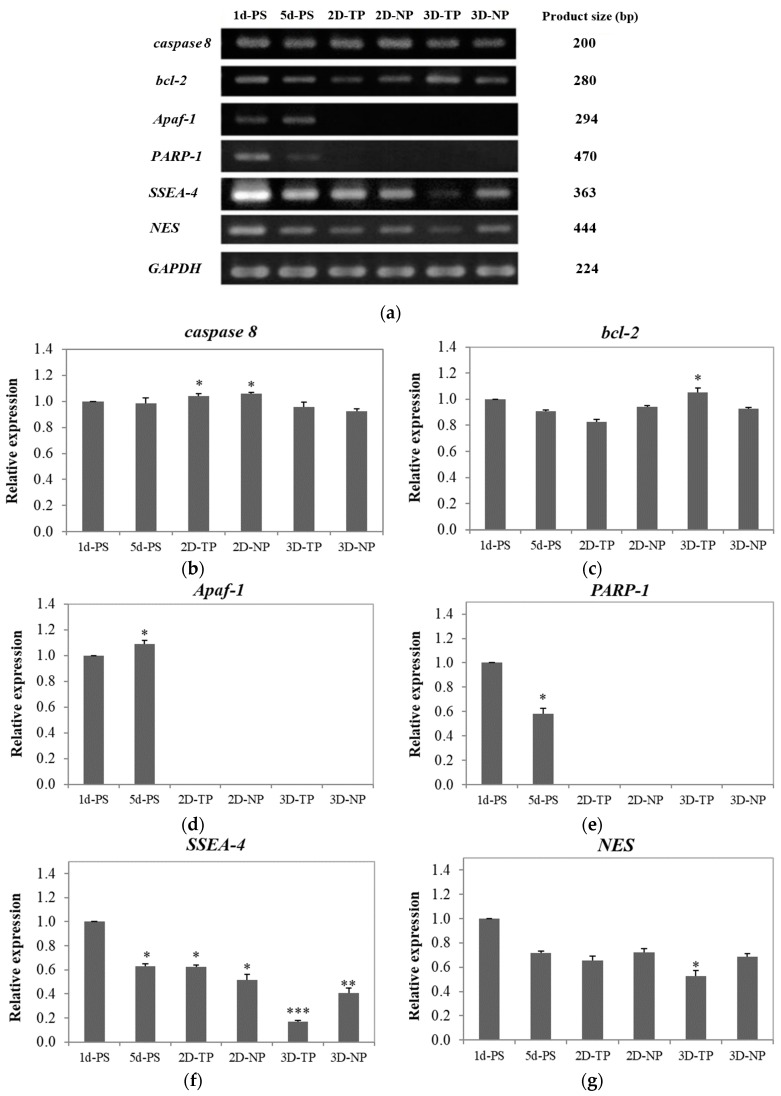
(**a**) Polymerase chain reaction (PCR) products were electrophoresed on agarose gels. The PCR product size of each gene is shown in the right column. Band intensity was used as a semi-quantitative measure of gene expression level after five days in culture and calculated to relative expression of the apoptosis/necrosis-related genes; (**b**) *caspase 8*, (**c**) *bcl-2*, (**d**) *Apaf-1*, and (**e**) *PARP-1*, and the pluripotency/differentiation-related genes—(**f**) *SSEA-4* and (**g**) *NES*. The asterisks (*, **, ***) on the top of the bars represented significant differences (*p* ≤ 0.05) in the relative expression of each gene among the scaffolds in comparison to 1d-PS.

To evaluate whether the scaffolds could support cell survival, the expression levels of multiple apoptosis/necrosis-related genes were investigated by RT-PCR and visualized by gel electrophoresis (Figure 10a). The apoptotic-related genes caspase 8 and *bcl-2* were detectable in all cultures (Figure 10b,c). Co-expression of *caspase 8*, *Apaf-1*, and *PARP-1* was found in 1d-ctrl and other 5d-ctrl (Figure 10b–d). On 2D-PCL scaffolds, pro-apoptotic *caspase 8* expression was higher than anti-apoptotic *bcl-2* expression, while cultures on 3D-PCL scaffolds expressed slightly more *bcl-2* than *caspase 8* (Figure 10b,c).

#### 3.4.2. Pluripotency/Differentiation-Related Genes

The pluripotency/differentiation status of cells cultured on each type of substrate was also evaluated. The highest expression of the stem cell marker, *SSEA-4*, was observed in control cells and the lowest level in cells growing on 3D-TP scaffolds. The rank order of expression was 5d-ctrl* = 2D-TP* ≥ 2D-NP** ≥ 3D-NP*** (Figure 10f). The control cultures also exhibited the highest expression level of the neural progenitor marker *NES*. Overall rank order was 5d-ctrl = 2D-NP > 2D-TP = 3D-NP and 3D-TP* (Figure 10g). Expression levels of the chondrocyte marker, *COL2A1*, and adipocyte marker, *PPAR-2* were undetectable in all cultures (data not shown).

## 4. Discussion

Polycaprolactone synthetic polymer was introduced more than a decade ago and has since been used extensively as a biomaterial for tissue engineering and regenerative medicine due to its advantageous properties, such as non-cytotoxicity and tunable biodegradability [1,15]. Moreover, many laboratories worldwide have continued to improve the biocompatibility of PCL, for instance by increasing its hydrophilicity [2,3,4]. The alkaline hydrolysis and low-oxygen plasma modified surface were applied to both 2D and 3D PCL scaffolds. In the physicochemical property evaluation, the commercial PS, the oxygen plasma-treated surface, was introduced as a control substrate. The results revealed that an alkaline hydrolysis PCL cannot affect the surface roughness observed from 2D-NP in comparison to PS, but might enhance the hydrophilicity of the PCL. Previous studies reported that the PCL-treated alkaline solution only improved surface wettability but did not change surface morphology [15,16]. Therefore, the oxygen-plasma treatment might play an important role in alteration of surface roughness and also increased hydrophilicity observed from 2D-TP and 3D-TP PCL scaffolds (Figure 3 and Figure 4). In addition, both plasma-treated PCLs were also increased more % O/C ratio (Figure 5), which could relate to increase their hydrophilicity (*p* ≤ 0.05). The oxygen plasma added some polar functional groups, –C–O–, –C=O, and –COOH, on the PCL surface [17], consequently resulting in better cell attachment and proliferation [1,4,18]. Furthermore, the geometric form (2Ds or 3Ds) of the biopolymer also impacted hydrophilicity. The 2D-NP and 2D-TP exhibited more hydrophilicity than the 3D-NP and 3D-TP, respectively (*p* ≤ 0.05) (Figure 4). The 3Ds expressed more surface roughness, peak density, and distribution than the 2Ds (Figure 3), which led to a decrease in focal contact area on the surface, and, thereafter, delayed water absorption was observed [21].

In this study, we found that there are substantial differences in proliferation, differentiation, and survival of the hWJMSC line, depending on the conformation (2Ds or 3Ds), surface roughness, hydrophilicity, and % O/C ratio. These results suggest that PLC is a flexible substrate for multiple applications in regenerative medicine and tissue engineering. The viability of attached cells, an important factor, definitely indicates the cytotoxicity and biocompatibility of novel materials [20,22]. Previous studies showed a non-cytotoxicity of PCL scaffolds either in 2D or 3D forms by maintenance proliferation and functions of various cell types, such as porcine chondrocytes [19], rat hippocampus [23], human bone marrow mesenchymal stem cells [24], and mouse embryonic stem cells [1]. Several methods to estimate the cell number, cytotoxicity, and cell viability, using microscopic examination, colorimetric, and chemiluminescent quantification assays, were suggested elsewhere [25]. The 2D and 3D PCL used in this study were opaque and absorbed the cell-staining dye, leading to difficulty in evaluating the cell number and viability by such methods mentioned in other reports. Therefore, in this case, the amount of cDNA was synthesized from the total RNA content, an alternative way to evaluate the cell viability [26]. As RNA is essential for the cell survival and mainly participates in many cell functions, including RNA processing and protein translation. The results of the viability assay (Figure 6) showed that the 3Ds gave the highest number of viable cells on day one, then started to decrease on day three, and noticeably dropped to the least on day five of culture (*p* ≤ 0.05), while, on the 2Ds culture, the cell viability continually increased from day one and reached to the highest on day five (*p* ≤ 0.05). Trends of viable cell number on either 2Ds and 3Ds seemed to relate to FAK and cyclin-A expression (Figure 7), the attachment and proliferation key proteins used in this research, respectively. The non-receptor tyrosine kinase FAK strictly required for cell−extracellular matrix (ECM) interactions during substrate adhesion [27]. The survival and proliferation of anchorage-dependent cells require FAK as a responsive mediator of integrin signaling [28,29]. FAK and other associated signaling pathways were demonstrated to regulate the cell cycle via cyclins and cyclin-dependent kinases (Cdks) [30,31]. Cyclin-A was chosen as a protein marker for cell proliferation capacity in the current study as it is a vital component of cell cycle machinery [32,33]. FAK expression was significantly higher in cells on 2Ds (*p* ≤ 0.05) (Figure 7) due to the fact that their surface roughness was less than that of the 3Ds, which provided more cell-substrate contact area for the focal adhering and spreading [31,32,33], hence leading to lower FAK expression on 3Ds (*p* ≤ 0.05) (Figure 7). However, in a stressful environment, after initial seeding (such as growth on the stiffer ECM under lower ligand stimulation), alternative signaling via RhoA protein to control the cell cycle through regulation of cyclin proteins may occur [28,30,31,32,33,34,35]. Recent reviews also indicated that the mesenchymal stem cells (MSCs) on 3D cultures may grow in the aggregated form, which can increase viability, proliferation, and paracrine effects by themselves [36]. Although there was no statistical difference between plasma-treated and non-plasma-treated scaffolds, slightly more FAK and cyclin A expressions can be observed on the plasma-treated and non-plasma-treated scaffolds, respectively (Figure 7).

The biocompatibility of PCL for cell–cell and cell–matrix interactions has been confirmed [37,38]. The surface microtopography of materials directly influences attachment patterns, cell shape, and spread [7,39]. According to the SEM (Figure 8), 2D scaffolds appeared to accelerate the initial rate of cell attachment. It is speculated that the surface of the 2D scaffolds provided anchorage points for initial attachment to hold the cell body in place, allowing subsequent filopodial protrusion to regulate cell shape and further growth [40]. Indeed, more numerous filopodial structures on 2D PCL scaffolds (Figure 9) have been shown to promote greater cell attachment [41,42]. This result was further verified by the increase of FAK expression described here (Figure 7), as filopodial shafts contain signaling pathways (e.g., FAK, Rac1/Cdc42/RhoA) promoting substrate adhesion [43]. Some studies have revealed that, in 3D environments, cells generally migrate into the substrate by producing various protrusion types, including some large adhesion patches called lamellipodia [41]. In fact, newly seeded cells transiently expressed filopodia for early attachment, which quickly disappeared [44] or converted into lamellipodia [40]. When migrating cells reach new environments, filopodia protrusion is initiated to detect surface topography, as obviously found on 3D-NP scaffolds (Figure 9). The 3D-TP surface provided higher oxygen and carbon atoms, which could facilitate in a focal contact, and, therefore, less filopodial protrusion was observed. These protrusions were then converted into lamellipodia and direct cell shape and spread [45,46,47]. Fewer filopodial protrusions and the conversion to lamellipodia may have conferred the irregular cell shape and undefined cytoplasmic edges observed in 3D PCL scaffold culture (Figure 9). Herein, there could be assumed that the configuration (2D or 3D) and plasma-treated PCL scaffold apparently affected hWJMSCs adhesion and migration behavior.

Cytotoxicity and viability testing are essential for biomaterial development but provide insufficient information for actual medical applications. In addition, other biological properties of the scaffolds have to be addressed in order to understand how they affect the target cells. Some scaffolds offer proper surface topography for cell adherence, but not proliferation, which leads to cell death [47,48]. There are two major types of cell death, apoptosis and necrosis, each governed by distinct regulatory mechanisms and containing unique biological and morphological manifestations [49]. Apoptosis or programmed cell death (PCD) is a key mechanism for embryogenesis and homeostasis in multicellular organisms. In contrast, spontaneous necrosis or cell lysis is a cellular response to acute nonphysiological injuries [50]. Initiation of either programmed apoptosis or necrosis is induced by external stimuli through a death ligand that subsequently activates a signaling cascade, including caspase family proteins. Programmed death appears to follow three routes: (1) *caspase 8* activations by external death signals cleaves an effector caspase (such as *caspase 3*), leading to apoptosis through an extrinsic pathway [51]; (2) mitochondria-mediated release of cytochrome C initiates apoptosome formation via the apoptotic protease activating factor, *Apaf-1*, activating the intrinsic apoptotic pathway [52,53]; and (3) external death signals stimulate *PARP-1*, resulting in programmed necrosis distinct from caspase-independent PCD [52,54]. Whether cells survive or die under specific conditions is determined by the balance in expression levels of competing gene groups involved in cell-fate decisions [55]. 

The expression of *caspase 8* was detectable in all culture samples (Figure 10b), but this does not necessarily indicate apoptosis without co-expression of other downstream pro-apoptotic factors (e.g., *Apaf-1* or *PARP-1*). Co-expression of *caspase 8*, *Apaf-1*, and *PARP-1* was found in 1d-PS and 5d-PS (Figure 10b,d,e), suggesting that some cells were undergoing apoptosis via an intrinsic pathway and/or necrosis pathway via activated *caspase 8/Apaf-1* [53,54] and *PARP-1*, respectively [51,55]. On 2D-NP and 2D-TP cultures, *caspase 8* expression was higher than *bcl-2* expression (*p* ≤ 0.05) (Figure 10b,c), suggesting in an extrinsic apoptosis pathway activation. The viable cells on those scaffolds rose to the highest number on day five (from the cell viability test), and this could lead to growing area limitation. Cultures on 3D PCL scaffolds expressed slightly more *bcl-2* than *caspase 8*, which suggests that most cells were able to grow and survive due to the anti-apoptotic actions of *bcl-2* [56]. In addition, the MSCs culture on 3D scaffolds usually displayed a better survival rate and higher resistance to oxidative stress-induced apoptosis via upregulation of *bcl-2* [57]. This evidence also supported the lowest *bcl-2* expression on 2D-TP culture where the oxygen atom on the surface increased by the plasma treatment. The more attached cells on 2D-TP, the more oxygen species interaction, leading to increase in oxidative stress, which eventually induced apoptosis. However, there was an exception for the commercial PS, since there was not only an oxygen atom attached to the surface but also nitrogen and other functional groups supporting cell growth [58]. 

Numerous specific combinations of stem cells and biomaterials have been examined to improve the intrinsic properties of scaffolds and understand how they influence proliferation and differentiation [57,58,59]. In this study, *SSEA-4* was chosen as a gene marker for stem cell self-renewal, while *NES*, *COL2A1*, and *PPAR-2* genes were used to assess the differentiation of neural, chondrocyte, and adipocyte lineages, respectively [59]. Co-expression of pluripotency and differentiation markers (*SSEA-4* and *NES*) observed in 1d-PS and 5d-PS (Figure 10f,g), which exhibited an equal level of relative expression to 2D-NP and 2D-TP, may result from the specific culture conditions employed. The presence of bFGF in the culture medium can induce *NES* expression (as well as the expression of other neural markers) without changing cell morphology [60,61]. SSEA-4-positive mesenchymal stem cells (MSCs) can be induced to either neural or non-neural lineages [62]. Alternatively, the cells may be in an intermediate state, between multipotent and differentiated states [60]. In addition, the differentiation capacity of cells is limited by density. A high density of hWJMSCs at confluence reduces proliferation rate, decreases colony-forming capacity, and ultimately leads to the loss of multi-potency [63]. Hence, 1d-PS, 5d-PS, 2D-NP, and 2D-TP were enriched in bFGF at low cell density, and so expressed the highest levels of *SSEA-4* and *NES*. With time in culture and concomitant proliferation, cells may have consumed most bFGF from the medium. Thus, by the time cells reached confluence on day five, there was a decrease in *SSEA-4* and *NES* expression in all samples.

For 3D PCL scaffolds, there were differences in the expression level of *SSEA-4* and *NES* between 3D-NP and 3D-TP. The 3D-NP culture expressed a higher level of *SSEA-4* and *NES* (Figure 10f,g), assuming that the 3D-NP exhibited a smoother surface with RMS roughness, ranging from 6.26 to 200 nm, which helps in facilitating neurons’ growth and enhancing Ca^2+^ accumulation [64,65]. This information also supported the co-expression of *SSEA-4* and *NES* in PS and 2D PCL, as described above.

The lowest expression levels of *SSEA-4* and *NES* expression were observed in cells on 3D-TP (Figure 10f,g). There were some possible explanations for this phenomenon: (1) the cells may reach confluence on day five, supported by a decrease in viable cells, resulting in the loss of proliferation capacity and/or self-renewal (stemness), due to the 3D-TP providing physicochemical guidance, which promotes cell proliferation rapidly [65]. (2) The irregular shape and aggregated form of cells were found on the surface, and this has been related to cell maintenance itself in a quiescent or inactive stage, also delayed replicative senescence [66,67]. The aggregated form of cells was not found only in 3D-TP; all substrates used in this study contained a high density of the cells at a confluent stage (data not shown). The previous microarray study revealed that downregulation of various differentiation-related genes, including chondrocyte-related and cytokine-related adipocyte differentiation genes, can be found in such cell shape [68]. This might be another piece of evidence supporting the absence of *COL2A1* and *PPAR-2* expression in this study. In addition, long-term culture of MSCs in medium supplemented with bFGF induced loss of osteogenic/adipogenic differentiation potential [63], while adding bFGF to a chondrocyte precursor culture, inhibited chondrocyte terminal differentiation [69] and osteogenesis of adipocyte-derived stromal cells in a dose-dependent and irreversible manner [70]. Inclusion of bFGF in the culture medium also downregulated the expression of adipogenic genes, thereby inhibiting adipogenesis and lipid accumulation [70,71,72]. Moreover, induction of *COL2A1* and *PPAR-2* expressions was required for the specific culture medium, growth factors, and long-term induction (approximately 14–21 days) [73,74]. 

The results revealed that stem cell activities were dependently manipulated by scaffold properties, including substrate, stiffness, and the degree of hydrophobicity. Surface modification obviously altered the scaffold properties. Alkaline treatment increased in surface hydrophilicity, while the plasma treatment modified overall surface properties, including RMS roughness and oxygen/carbon ratio obtaining varied PCL scaffolds characteristics. The 2D PCL scaffolds provided the focal contact area and preferable surface properties for cell adhesion and proliferation, and also modulated the gene expression, which was identical to the commercial PS. Since the 3D PCL enhanced cell proliferation, migration, and apoptosis protection, the cells might stay in the quiescence stage, especially in 3D-TP. However, an in vivo can be performed, in order to study and gain a better understanding of cell–substrate interactions in the body system.

## 5. Conclusions

This research was the first trial to evaluate the intrinsic biological responses of hWJMSCs cultured on alkaline hydrolysis and low-pressure oxygen plasma modified 2D and 3D PCL scaffolds, and without adding any differentiation inducers. Both 2D and 3D PCL scaffolds enhanced cell attachment and proliferation compared to a commercial substrate (PS) but slightly altered the stemness properties. The 2D scaffolds appeared to be more suitable for rapid cell adherence and propagation, while the 3Ds offered greater opportunities for cell migration and anti-apoptosis/necrosis. Therefore, the 3D-TP would be used as a substrate for stem cell homing and accommodating in cell delivery system for tissue repair and endogenous regenerative medicine.

## Figures and Tables

**Table 1:** Primer sequences and accession numbers.

Gene	Accession Number	Sequence
*caspase 8*	NM_001228.4	F: 5′-TGCAGGGGCTTTGACCACGA-3′ R: 5′-TGGGGGCCTCCTGTCCATCA-3′
*bcl-2*	NM_000633.2	F: 5′-TGTGGCCTTCTTTGAGTTCG-3′ R: 5′-TCACTTGTGGCTCAGATAGG-3′
*Apaf-1*	NM_013229.2	F: 5′-TGGCCAGTGCCAAGATGCACA-3′ R: 5′-CGACCTCCTGCTTGGCCTGC-3′
*PARP-1*	NM_017915.3	F: 5′-AATCTCCAGGGGGTAGAACT-3′ R: 5′-CAGAGCCTGTTGAAGTTGTG-3′
*SSEA-4*	NM_203289.4	F: 5′-GCCCTAGAACTCCAATCACA-3′ R: 5′-CCCAGATGGTATTGGACACA-3′
*NES*	NM_006617.1	F: 5′-TCCTGCTCGCTCTCTACTTT-3′ R: 5′-CCCAGATGGTATTGGACACA-3′
*COL2A1*	NM_001844.4	F: 5′-CCCATTGGTCCTTGCATTAC-3′ R: 5′-GTCCTCTGCGACGACATAAT-3′
*PPAR-2*	NM_138712.3	F: 5′-GCATTATGAGACATCCCCACT-3′ R: 5′-CCTATTGACCCAGAAAGCGAT-3′
*GAPDH*	NM_002046.4	F: 5′-TGCTGGCGCTGAGTACGTCG-3′ R: 5′-TGACCTTGGCCAGGGGTGCT-3′
